# Transfection by Electroporation of Cancer and Primary Cells Using Nanosecond and Microsecond Electric Fields

**DOI:** 10.3390/pharmaceutics14061239

**Published:** 2022-06-11

**Authors:** Eivina Radzevičiūtė, Veronika Malyško-Ptašinskė, Jurij Novickij, Vitalij Novickij, Irutė Girkontaitė

**Affiliations:** 1 State Research Institute Centre for Innovative Medicine, Department of Immunology, 08406 Vilnius, Lithuania; irute.girkontaite@imcentras.lt; 2 Faculty of Electronics, Vilnius Gediminas Technical University, 03227 Vilnius, Lithuania; veronika.malysko-ptasinske@vilniustech.lt (V.M.-P.); jurij.novickij@vilniustech.lt (J.N.)

**Keywords:** plasmid DNA, transfection, electroporation, GFP, luciferase, CHO-K1, 4T1, DC

## Abstract

Gene transfer into primary immune cells as well as into cell lines is essential for scientific and therapeutical applications. One of the methods used for gene transfer is electroporation (EP). EP is a method where a pulsed electric field (PEF) causes a highly transient permeability of the targeted cell membrane. In this work, we present the electrotransfection of CHO-K1, 4T1 cell lines, and primary murine DCs with detectable protein-encoding plasmids in the sub-microsecond range. Microsecond (µs)- and nanosecond (ns)-range pulsed electric field transfection protocols were used. The efficiency of electrotransfection was evaluated using green fluorescent protein (GFP)-encoding plasmids (4.7 kbp; p-EGFP-N1) and plasmids expressing a firefly luciferase and red fluorescent protein (tdTomato) (8.5 kbp; pcDNA3.1(+)/Luc2 = tdT)). It was shown that the used nsPEFs protocol (7 kV/cm × 300 ns × 100, 1 MHz) ensured a better transfection efficiency than µsPEFs (1.2 kV/cm × 100 µs × 8, 1 Hz). Plasmid size and concentration had a strong impact on the cell transfection efficiency too. We also showed that there were no significant differences in transfection efficiency between immature and mature DCs. Finally, the nsPEF protocols were successfully applied for the stable transfection of the CHO-K1 cell line with the linearized pcDNA3.1(+)/Luc2 = tdT plasmid. The results of the study are applicable in gene therapy and DNA vaccination studies for the derivation of optimal electrotransfection conditions.

## 1. Introduction

The genetic modification of cell lines and primary cells often involves the use of viral vectors. However, one of well-known forms of nonviral transfection is electroporation (EP) [1,2]. EP is a method where a pulsed electric field (PEF) induces an increase in the affected cell membrane permeability to deliver charged molecules into the cells [1,3]. A unique advantage of electroporation compared to viral vectors is that this method can be used for both oligonucleotides and proteins transport [4]. To generate a high-intensity PEF, a voltage is usually applied between the two electrodes surrounding the cellular medium and the generated electric field strength influences the transmembrane potential induction over the cellular membrane. PEF treatment is applied between the two electrodes, and the generated electric field strength influences the transmembrane potential differences over the cellular membrane. Aqueous pore formation in the cell membrane occurs when the transmembrane potential difference exceeds a critical value. If that exposure is sufficiently short and the membrane recovers rapidly enough for the cell to remain viable, electroporation is called reversible, otherwise, it leads cells to death and is described as irreversible EP [3,5]. Reversible electroporation and high cell viability are crucial for efficient cell transfection.

It is well known that EP in vitro is efficient in a broad variety of prokaryotic as well as mammalian cell lines. However, each type of cell requires different electroporation conditions to ensure efficient transfection. In the literature, the main parameters affecting the effectiveness of electroporation include electric field strength, number of pulses, duration of pulse, and the frequency applied. In addition, temperature, conductivity of the electroporation medium, molecular size, concentration of the transported molecules, shape of the target cells, charge of the cell membrane, as well as the cell density affect the electroporation [6,7,8,9,10,11,12,13]. In a variety of studies, micro- or nanosecond pulses were applied to widely used mammalian hosts—Chinese hamster ovary (CHO) cells [8,9,10,14,15,16], and to evaluate the efficacy of a gene electrotransfection, different protein-encoding plasmids were used. However, one of the most commonly used plasmids are green fluorescent protein (GFP)-encoding plasmids (3.5 kb, pMAX-GFP [12,14,16,17,18]; 4.7 kb, pEGFP-N1 [14,15]) and luciferase-expressing plasmids (4.6 kbp pGL4.13 [16,19]). The use of model cell lines such as CHO and standard plasmids is a common practice to improve the repeatability of the results during derivation of the optimal electroporation conditions. 

Parameter-wise, long micro–millisecond-range electric field pulses such as the European Standard Operating Procedures for Electrochemotherapy (ESOPE) have been used for decades [9,10,20]. It is a safe and effective procedure; however, microsecond protocols can be associated with undesirable side-effects such as muscle contractions in vivo or electrochemical reactions negatively affecting plasmid DNA [20,21]. For that reason, studies of the application of shorter, nanosecond pulsed electric fields (nsPEFs) on different biological systems are needed. Guo et al. [22] showed that the pre-treatment with nsPEF significantly increased the transfected cell viability and enhanced gene expression. In contrast, another study concluded that nsPEFs combined with standard µsPEF parameters had no effect on gene electrotransfer [15]. Recently, we presented a proof of concept [23] and sub-microsecond-range electrotransfection protocols of CHO-K1 cells, which resulted in a high transfection efficiency and cell viability [14]. It is a developing field requiring more applied research to further support the feasibility of nsPEFs in electrotransfection.

At the same time, the application of model cell lines and model plasmids does not necessarily mean good repeatability of the protocols when used with another cell line. For example, dendritic cells (DCs) are a diverse group of leukocytes. DCs are the professional antigen-presenting cells (APCs), and DCs cross-present antigens to both CD4+ and CD8+ T cells, which play critical roles in regulating the innate and adaptive immune response. DCs exist primarily in two basic functional states: immature (iDC) and mature (mDC) [24,25]. iDCs reside in the tissue microenvironment, where they survey for incoming foreign pathogens. Pathogens that have entered the body activate DCs, which leads to migration to the draining lymph node, secondary lymphoid organs, where they trigger a specific tumor-reactive T cell response [26] In vitro DCs are activated to mature by the inflammatory cytokines TNF-α and IL-1, by LPS, and by the CD40 ligand (CD40L) [27]. In the last two decades, there has been immense progress in personalized combined immunotherapies for cancer treatment. Due to the DC’s antigen presentation ability, they have a major role in cancer immunotherapies. DCs are used as vaccine platforms to induce anti-tumor immune responses [26]. In dendritic cell vaccine studies, DCs were electroporated with the GFP to compare RNA-encoded tumor-associated antigens (TAAs) transfer [11,28,29] or to track loaded DCs in vivo. The electrotransfection of murine bone marrow dendritic cells (BMDCs) with pEGFP-N1 reached only 1–2% of GFP-positive cells, which has been explained by the apoptosis of electroporated cells [30].

Another study revealed that the GFP mean fluorescence intensity (MFI) of mature DCs remained twofold higher than the MFI of immature DCs [31]. In contrast, Michiels et al. concluded that there is no significant difference in transgene-expression DCs electroporated either before or after maturation [28]. Still, most primary hematopoietic cells, including BMDCs, are still refractory to conventional electrotransfection, and the model cell lines such as CHO-K1 cannot be used to predict the electrotransfection efficiency. 

Another application of electrotransfection methods can be highlighted in the context of in vivo cancer biology studies and noninvasive imaging technologies of living bodies. Fluorescent imaging uses fluorescent proteins and dyes, while bioluminescence uses luminescent enzymes [32]. Such methods allow the detection of signals in deep tissues of small living animals, which is useful for cancer research, to evaluate tumor size as well as the kinetic development of metastasis [33]. Therefore, the development of cancer cell lines expressing fluorescent or bioluminescent proteins has a high actuality and higher flexibility compared to microscopic methods used for metastasis evaluation at the end of the experiment [32,34,35].

The purpose of this study was to detect the differences in electrotransfection efficiency between CHO-K1, 4T1 cell lines, and primary murine APCs, with detectable protein-encoding plasmids. Microsecond (µs)- and nanosecond (ns)-range PEF transfection protocols were compared. The differences in response to various cell lines under the same treatment conditions were of utmost interest. We used two different sizes and different proteins-encoding plasmids (4.7 kbp p-EGFP-N1; 8.5 kbp pcDNA3.1(+)/Luc2 = tdT). First, we evaluated the permeabilization of CHO-K1, murine mammary carcinoma (4T1), and primary murine DCs using µsPEF and nsPEF protocols. After that, we determined the transfection efficiency with different concentrations of those two plasmids and selected the best plasmid concentrations for electrotransfection of the 4T1 cell line and primary murine DCs. We also studied the primary DC’s transfection efficiency using immature and mature DCs. For DC maturation, LPS and TNF-α were used. 

It was shown that electroporation with nsPEF protocols ensured equivalent or even better transfection efficiency than µsPEF. In addition, plasmid size had a high impact on CHO-K1, 4T1, and the primary DC’s transfection efficiency. Lastly, it was shown that different cell types affect the transfection efficiency dramatically despite the similar susceptibility to electroporation.

## 2. Materials and Methods

### 2.1. Electroporation Setup and Parameters

The experimental setup consisted of a 3 kV, 100 ns–1 ms square-wave high-voltage pulse generator (VilniusTECH, Vilnius, Lithuania) [36], and a commercially available electroporation cuvette with a 1 mm gap between electrodes (Biorad, Hercules, CA, USA). The voltage applied to the cuvette was 0.12, 0.7 kV, corresponding to a 1.2–7 kV/cm electric field, respectively. For µsPEF, the (1.2 kV/cm × 100 µs × 8, 1 Hz) protocol was used. The nsPEF involved sequences of 100 pulses delivered at 1 MHz frequency (7 kV/cm × 300 ns × 100).

### 2.2. Cell Culture

Chinese Hamster Ovary cells CHO-K1 were maintained in RPMI 1640 medium supplemented with glutamine, 100 U/mL of penicillin, 100 mg/mL of streptomycin, and 10% of fetal bovine serum (FBS). Murine mammary carcinoma cell line 4T1 was grown in RPMI 1640 medium supplemented with 10% FBS, glutamine, 25 mM HEPES, 100 U/mL of penicillin, and 100 μg/mL of streptomycin. All cell culture reagents were obtained from Gibco, Thermo Fisher Scientific, New York, NY, USA. The cells were cultured at 37 °C, 5% CO_2_. 

The day before electroporation, the CHO-K1 and 4T1 cells were plated into 6-well tissue culture plates, 0.5 × 10^6^ cells per well. The next morning, the cells were trypsinized (Thermo Fisher Scientific, Waltham, MA, USA), centrifuged, and resuspended in the buffer for electroporation (242 mM saccharose, 5.5 mM Na_2_HPO_4_, 4 mM NaHPO_4_, 1.7 mM MgCl_2_, pH 7.1) at a concentration of 6 × 10^6^/mL. For the viability assay, cells were resuspended in the electroporation buffer at concentration of 2 × 10^6^/mL.

The day before electroporation, DCs were kept to ensure sufficient cell confluence, and the next morning, they were gently removed. DCs were resuspended in the same electroporation buffer at a concentration of 10 × 10^6^/mL, and for the viability assay, at a concentration of 4 × 10^6^/mL. All the samples were incubated on ice for 20 min before electroporation.

### 2.3. In Vitro Generation of Bone Marrow Dendritic Cells (BMDCs)

Murine BMDCs were produced based on existing protocols [37,38,39]. Six- to eight-week-old female BALB/c mice were euthanized by cervical dislocation. Bone marrow was isolated from mice by carefully dissecting tibias and femurs, after those bones were cleaned and transferred in sterile phosphate-buffered saline (PBS) solution. All other work was performed inside a biosafety hood to avoid contamination. For disinfection, the bones were placed in ethanol 70% for 30 s and then again in sterile PBS. Afterward, the ends of the bones were cut, and 1 mL of sterile PBS was infused inside of the bone using a sterile syringe. Collected suspension was washed and cells were cultured in the presence of 5% murine granulocyte-macrophage colony-stimulating factor (GM-CSF; home-made) at a maximum density of 750 × 10^3^ cells/mL in RPMI 1640 medium with 2 mM glutamine, 100 U/mL of penicillin, 100 mg/mL of streptomycin, and 10% of FBS. After 3 days, fresh medium with GM-CSF was added. At the 5th day, a part of the immature DCs was incubated for 24 h with LPS (0.1 µg/mL) (Sigma-Aldrich, St. Louis, MO, USA) and TNF-α (200 U/mL) (Thermo Fisher Scientific, Waltham, MA, USA).

All experimental protocols were approved by the Lithuanian State Food and Veterinary Service (approval G2-145) and the study was carried out in strict accordance with the recommendations in the Guide for the Care and Use of Laboratory Animals.

### 2.4. Cell Permeabilization

Cell permeabilization efficiency was evaluated using Green-fluorescent stain Yo-Pro1 (Sigma-Aldrich, St. Louis, MO, USA) and BD Accuri C6 flow cytometry (BD Biosciences, San Jose, CA, USA). The cells in the electroporation buffer (same as in electrotransfection) were mixed with Yo-Pro1 for the final concentration of 1 μM. The 50 μL samples were placed between the electrodes and treated with different PEF protocols followed by 3 min of incubation at room temperature; after that, 150 μL of PBS was added and samples were analyzed by flow cytometry. Yo-Pro1 (491⁄509) fluorescence was detected through Channel FL1 (533/30 nm BPF). The results were analyzed by FlowJo (BD, Franklin Lakes, NJ, USA).

### 2.5. Cell Electrotransfection

**Figure 1:**
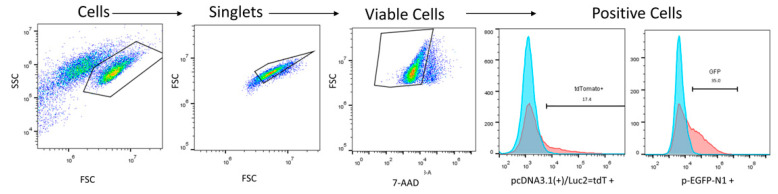
A representative nested gating strategy illustrating transfected cells analysis.

Cell electrotransfection efficiency was evaluated by using green fluorescent protein-encoding plasmids (4.7 kbp (p-EGFP-N1)) and plasmids expressing a firefly luciferase and red fluorescent protein (tdTomato) (8.5 kbp (pcDNA3.1(+)/Luc2 = tdT)). The pcDNA3.1(+)/Luc2 = tdT plasmid was generously provided by Christopher Contag [40]. Each electrotransfection was performed using 30 μL of ice-cold cell suspension and added plasmid DNA (p-EGFP-N1 or pcDNA3.1(+)/Luc2 = tdT) purified with the HiPure Expi Plasmid Gigaprep Kit (Thermo Fisher Scientific, Waltham, MA, USA). The final concentrations of 0.07, 0.2, and 0.3 μg/μL for each plasmid were used. Immediately after electroporation, the cells were transferred into a 48-well plate and incubated for 10 min on ice, followed by a 0.5 mL addition of the cell culture growth media. The cells were incubated for 24 h at 37 °C, 5% CO_2_. Afterward, CHO-K1 and 4T1 cells were detached from the well using trypsin, while DCs were gently removed by pipetting. Transfected cells were analyzed using a BD Accuri C6 flow cytometer. Viable cells for flow cytometry were determined by 7-amino-actinomycin D (7-AAD; Thermo Fisher Scientific, Waltham, MA, USA), which intercalates into double-stranded nucleic acids. The transfected cells fluorescence of 7-AAD, GFP, and tdTomato was detected using Channel FL3 (560 nm LP), FL1 (533/30 nm BPF), and FL2 (585/40 nm BPF), respectively. The gating strategy for electrotransfection analysis is presented in Figure 1.

The gate was defined based on untreated control. The viable cells within the gate were defined as GFP positive.

### 2.6. Plasmid Restriction

The pcDNA3.1(+)/Luc2 = tdT plasmid was restricted with PvuI enzyme. The digestion mix for 1 sample (20 μL) was as follows: 1 μg of DNA (2 mg/mL, 0.5 μL), 2 μL of buffer R, 15.5 μL of deionized water, 2 μL of PvuI restriction enzyme. The digestion mix with plasmid DNA was incubated for 4 h at 37 °C.

The linearized plasmid was concentrated by EtOH precipitation: 0.2 volumes of CH_3_COONa (3 M, pH 4.8) and 3 volumes of 96% EtOH were added. After 15 min of incubation on ice, the vial with precipitated DNA was centrifuged (31,840× *g*, 15 min). Afterward, the plasmid was washed with 70% EtOH and centrifuged again. The pellet was resuspended in sterile water. 

The DNA optical density (OD260) was measured with a multimodal plate reader. According to the formula OD260 × dilution × 0.05, the DNA concentration was calculated in mg/mL. The prepared plasmids (0.5 µg/µL) were used for stable transfection.

### 2.7. CHO-K1 Stable Transfection

A volume of 3 μL of linearized plasmid DNA was mixed with 27 μL of CHO-K1 cell suspension (6 × 10^6^/mL) prepared in RPMI media. The sample (30 μL) was transferred to the electroporation cuvette and pulsed electric fields were applied (nsPEF-7 kV/cm × 300 ns × 100 or µsPEF-1.2 kV/cm × 100 µs × 8, 1 Hz). After electrotransfection, 30 μL of the sample was transferred to a Petri dish where the cells were incubated for 10 min. After incubation, 10–12 mL of the cell culture medium was added. The Petri dish with cells was transferred to the incubator for further growth at 37 °C in 5% CO_2_ for 48 h.

After 48 h, 400 μg/mL of antibiotic G418 was added. After another 48 h, the concentration of antibiotic was increased up to 600 μg/mL. Only G418-resistant cells stayed alive and were cloned by transferring them into separate 96-wells. Then, clones were named and expanded (grown up to a week). Half of the cloned cells were transferred into another 96-well plate for the bioluminescence detection assay. The best CHO-K1 luciferase clones were selected for measurement of bioluminescence.

### 2.8. Bioluminescence Detection Assay

After 24 h, electroporated cells were transferred into the white 96-well plates. D-Luciferin (Promega, Madison, WI, USA) was added to the cells at a final concentration of 300 μg/mL. The luminescence of pcDNA3.1(+)/Luc2 = tdT plasmids expressing firefly luciferase was evaluated using a Synergy 2 microplate reader and Gen5 software (BioTek, Shoreline, WA, USA) for 120 min with a 10 min time step. For the stably transfected CHO-K1 cell line subclone bioluminescence activity, 0.02 × 10^6^ cells per well was used.

### 2.9. Viability Assay

The electroporation was performed as described above. Later, the cells after electroporation were transferred into 96-well flat-bottomed plates and incubated for 10 min on ice, followed by a 0.2 mL addition of the cell culture growth media. Additionally, the wells with non-transfected cells (PEF-untreated control) were prepared. The next day, 10 µL of the cell viability reagent (AlamarBlue, Thermo Fisher Scientific, Waltham, MA, USA) was pipetted into each well. The cells were kept in an incubator for the next 4 h and the fluorescence was measured using a Synergy 2 microplate reader and Gen5 software (BioTek, Shoreline, WA, USA).

### 2.10. Fluorescence Microscopy

A period of 24 h before microscopy, the transfected CHO-K1 cells were prepared. Cells were seeded in a Petri dish at a concentration of 2 × 10^5^ cells/mL and sterilized coverslips were added. After the incubation period, coverslips were washed with PBS and attached cells were stained using one drop of acridine orange (AO), before covering by a cover slip. 

The fluorescent signal was visualized by fluorescent microscopy using a Leica DMBL microscope (Leica, Wetzlar, Germany). To view the fluorescent tdTomato protein, a filter set with Ex = BP 515–560 and Em = LP 590 was used (×40 (CTRL) and ×100 (nsPEF; immersion)).

### 2.11. Statistical Analysis

One-way analysis of variance (ANOVA; *p* < 0.05) was used to compare different treatments. Tukey’s HSD multiple comparison test for the evaluation of the difference was used when ANOVA indicated a statistically significant result (*p* < 0.05 was considered as statistically significant). The data were post-processed using the GraphPad Prism 8 software program (GraphPad Software; La Jolla, CA, USA). Each experimental point was obtained from at least three independent experiments, and results are represented as mean ± standard deviation.

## 3. Results

### 3.1. PEF-Induced Electropermeabilization

**Figure 2:**
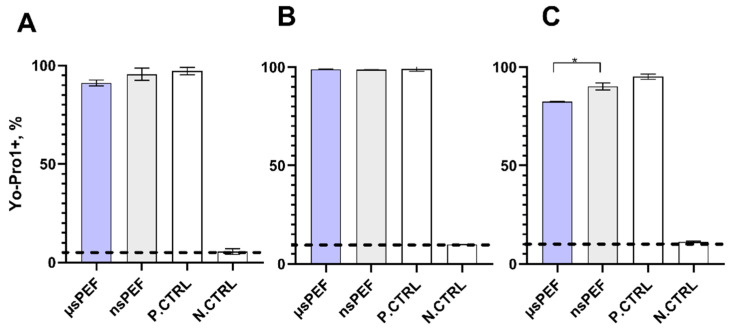
Cell permeabilization using µsPEF (1.2 kV/cm x 100 µs × 8 pulses) and nsPEF (7 kV/cm × 300 ns × 100 pulses (1 MHz)): CHO-K1 (**A**), 4T1 (**B**), and DCs (**C**). Positive control (P. CTRL)—(1.5 kV/cm × 100 µs × 8 pulses); negative control (N. CTRL)—nontreated cells. Asterisk (*) corresponds to statistically significant (*p* < 0.05) difference.

It is well known that a high permeabilization of the target cells must be reached to ensure efficient DNA transfer. Thus, we first electroporated CHO-K1, 4T1, and primary DCs, using microsecond (µs)- and nanosecond (ns)-pulsed electric fields. Targeted cell membrane permeabilization was determined by measuring the uptake of Yo-Pro1 by flow cytometry. The results are presented in Figure 2.

Both usPEF and nsPEF protocols used to induce permeabilization trigger high permeabilization of the CHO-K1 (Figure 2A), 4T1 (Figure 2B) cell lines, as well as murine DCs (Figure 2C). CHO-K1 and 4T1 cell lines while affected with microsecond and nanosecond pulses reached the saturated permeabilization (~90%). The percent of permeabilized cells and uptake of fluorescent dye were above 80% in every electroporated sample. The permeabilization efficiency of primary murine dendritic cells was slightly lower (~80%) compared to CHO-K1 and 4T1 cells when µsPEF was used (Figure 2C). However, in all cases, the permeabilization rates were high enough to perform further electrotransfection experiments.

### 3.2. Electrotransfection of CHO-K1 Cell Line

**Figure 3:**
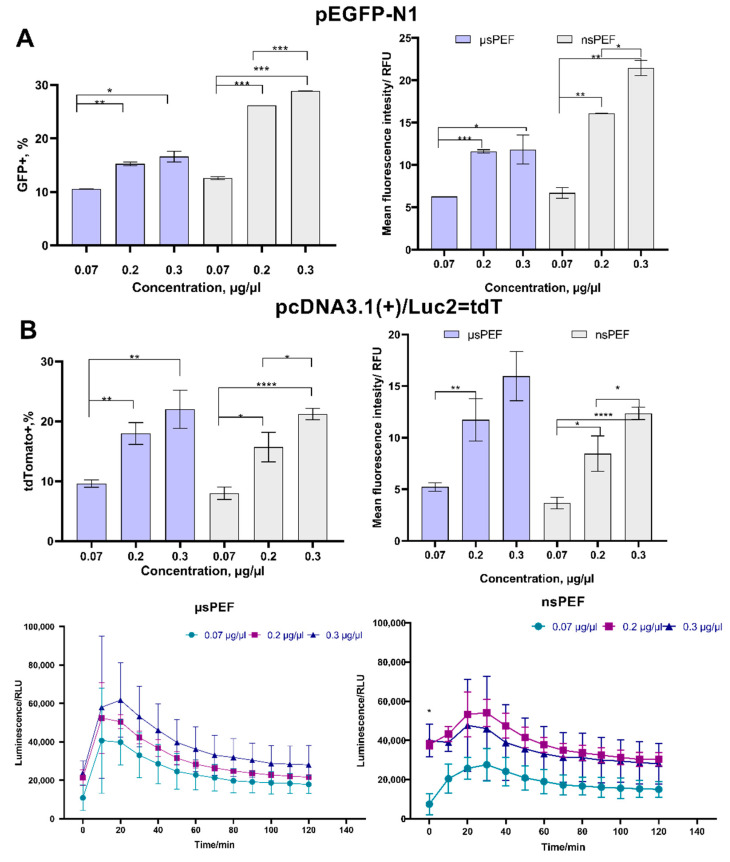
Transfection efficiency of CHO-K1 cell line during microsecond 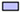 and nanosecond 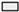 electroporation, using (**A**) pEGFP-N1 plasmid and (**B**) pcDNA3.1(+)/Luc2 = tdT plasmid. The MFI was normalized to the negative control. Asterisk (*) corresponds to statistically significant (* *p* < 0.05; ** *p* < 0.01; *** *p* < 0.001; **** *p* < 0.0001) difference.

To determine the optimal pEGFP-N1 and pcDNA3.1(+)/Luc2 = tdT plasmids concentration for microsecond (µsPEF) and nanosecond (nsPEF) protocols, the CHO-K1 cells were transfected using different concentrations of those plasmids (0.07, 0.2, and 0.3 μg/μL). After 24 h, the transfection efficiency was evaluated using flow cytometry for fluorescence and the microplate reader for bioluminescence. The percentage of GFP-positive cells and mean fluorescence intensity (MFI) are presented in Figure 3A. The TdTomato-positive cells percentage, MFI, and kinetic measurements of bioluminescence are shown in Figure 3B.

As can be seen in Figure 3A, a better transfection efficiency with GFP was reached using the nsPEF protocol (~30%; µsPEF~18%). The lowest transfection efficiency was reached when 0.07 μg/μL of pEGFP-N1 plasmid was used. No significant difference in transfection efficiency was observed when 0.2 and 0.3 μg/μL concentrations of plasmid were used with the µsPEF protocol. The nsPEF protocols resulted in a more pronounced difference (i.e., transfection efficiency was higher when the 0.3 μg/μL versus the 0.2 μg/μL protocol was used (*p* < 0.05)).

The CHO-K1 cell line transfection results with pcDNA3.1(+)/Luc2 = tdT plasmids can be seen in Figure 3B. The differences in transfection rates (evaluated based on fluorescence) were more profound when different concentrations of pcDNA3.1(+)/Luc2 = tdT plasmids were used, but nsPEF and µsPEF treatments returned comparable results. In addition, the pcDNA3.1(+)/Luc2 = tdT plasmid encodes firefly luciferase, which can be detected by bioluminescence kinetic measurements. As can be seen in Figure 3B, a plasmid concentration-dependent response was observed too. In the case of nanosecond pulses, 0.2 and 0.3 μg/μL concentrations of the plasmid returned similar transfection rates, which were significantly higher compared to that of the 0.07 μg/μL protocol. The fluorescent tdTomato protein-encoding plasmid was almost two times larger than the GFP-encoding plasmid (8.5 versus 4.7 kbp). Plasmid size had little effect on transfection efficiency during the µsPEF procedure, while a significantly higher transfection rate was achieved with a smaller plasmid when nanosecond sequences were employed. 

A further study was limited to the 0.2 μg/μL concentration of the plasmid due to minor differences in transfection efficiency when compared to the 0.3 μg/μL methodology.

### 3.3. Electrotransfection of 4T1 Cell Line

**Figure 4:**
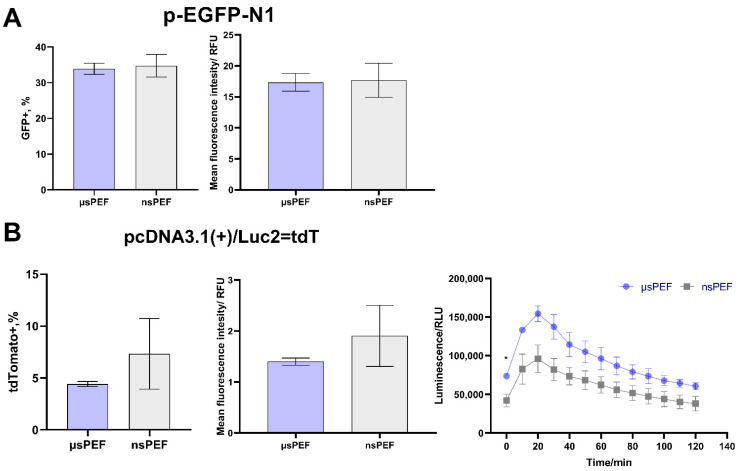
Transfection efficiency of murine 4T1 cell line during microsecond 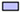 and nanosecond 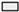 electroporation, using (**A**) pEGFP-N1 plasmid and (**B**) pcDNA3.1(+)/Luc2 = tdT plasmid. The MFI was normalized to negative control. Asterisk (*) corresponds to statistically significant difference (*p* < 0.05).

The 4T1 murine metastatic cell line transfection efficiency was further evaluated (Figure 4). The GFP-positive cells percentage and MFI are presented in Figure 4A, while the results with firefly luciferase and red tdTomato fluorescent protein are shown in Figure 4B.

As can be seen in Figure 4A, no significant differences between µs and ns PEFs protocols were observed in the transfected 4T1 cells with the pEGFP-N1 plasmid. The percentage of GFP-positive cells of more than 30% was reached. However, noticeable differences in response were observed when the 4T1 cell line was transfected with the pcDNA3.1(+)/Luc2 = tdT plasmid (Figure 4B). The percentage of fluorescent cells reduced more than sixfold for both protocols, which was not the case for the CHO-K1 cell line. Nevertheless, the bioluminescence signal was extremely high in the transfected cells. Contrary to CHO-K1 cell line transfection, higher bioluminescence intensities were reached with microsecond pulses.

Both cell lines (4T1 and CHO-K1) were transfected with the same pulse parameters and same plasmids; however, the differences in the responses were obvious. It further implies that the electrotransfection efficiency depends on a variety of parameters that must be determined for each cell line separately. 

### 3.4. Electrotransfection of Primary Murine DCs

**Figure 5:**
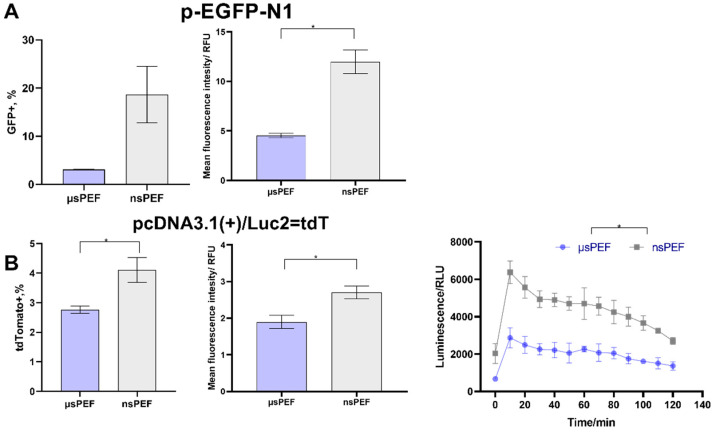
Transfection efficiency of primary murine DCs during microsecond 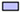 and nanosecond 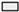 electroporation, using (**A**) pEGFP-N1 plasmid and (**B**) pcDNA3.1(+)/Luc2 = tdT plasmid. The MFI was normalized to negative control. Asterisk (*) corresponds to statistically significant (*p* < 0.05) difference.

We transfected immature primary DCs using the same methodology as described above. The results are shown in Figure 5. The percentage of GFP-positive DCs and MFI are presented in Figure 5A. There were significant MFI differences comparing microsecond and nanosecond pulse duration protocols. The MFI of cells transfected using nsPEFs was two-fold higher than the MFI of DCs transfected with µsPEFs.

The DC’s transfection efficiency with the pcDNA3.1(+)/Luc2 = tdT plasmid was further analyzed and the results are presented in Figure 5B. The percentage of tdTomato-positive cells was lower compared to the smaller GFP-encoding plasmid. Nevertheless, the same tendency of nsPEF being superior to microsecond procedures was still in place.

Although the same electrotransfection parameters and plasmids were used as with CHO-K and 4T1 cell lines, the transfection efficiency with primary DCs was significantly lower with both used plasmids. 

**Figure 6:**
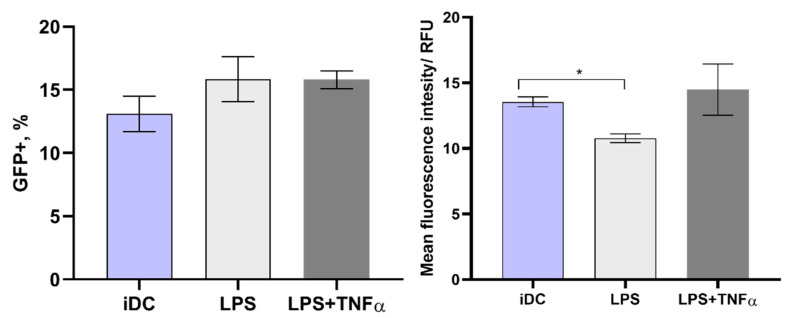
nsPEF electrotransfection efficiency of primary murine DCs. From the left, immature DC, LPS, and LPS and TNF-α maturated DC. MFI was normalized to negative control. Asterisk (*) corresponds to statistically significant (*p* < 0.05) difference.

According to the available scientific literature [28,31] and to enhance the DC’s transfection efficiency, experiments were also performed with both immature and matured DCs. Based on the acquired data on nsPEF protocol efficiency, the electrotransfection of iDCs and different maturated mDCs was performed only using the nsPEF protocol and the smaller pEGFP-N1 plasmid. The results are summarized in Figure 6. It can be seen that the electrotransfection efficiency of murine DCs did not improve compared with iDCs, irrespective of different maturation.

### 3.5. Viability Results

The viability of all three cell lines post-electrotransfection was evaluated after 24 h. The results are presented in Table 1. As can be seen in Table 1, the CHO-K1 cell line was not affected by PEF treatment. The loss of viability was not statistically significant (*p* > 0.05) versus control.

In the case of the 4T1 cell line, the viability dropped up to 30% when μsPEF protocols were used. A similar response was acquired with both plasmids. However, the nsPEF resulted in a better treatment. With a p-EGFP-N1 plasmid, the viability dropped less than 10%, while in the case of the larger pcDNA3.1(+)/Luc2 = tdT, the loss in viability was less than 20%. In both cases, the viability of the 4T1 cells was significantly (*p* < 0.05) higher when compared to μsPEF protocols. The DCs on average showed the lowest viability when treated by μsPEF; however, the response was comparable with 4T1 (*p* > 0.05). The nanosecond pulses triggered almost a 30% drop in DC viability with both plasmids, which, on average, was lower than μsPEF, but still not statistically significant. 

### 3.6. CHO-K1-Stable Transfection with pcDNA3.1(+)/Luc2 = tdT Plasmid

**Figure 7:**
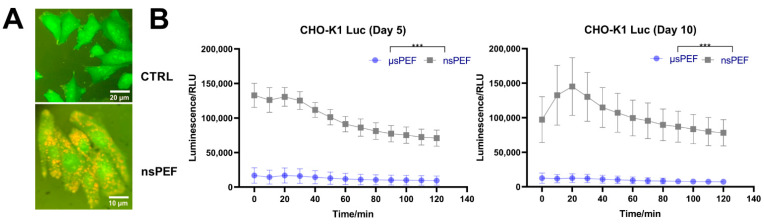
(**A**) Transfected CHO-K1 cells expressing tdTomato protein visualized using fluorescence microscopy (×40 (CTRL) and ×100 (nsPEF; immersion)). (**B**) Bioluminescent changes in selected CHO-K1 cell line clones 5- and 10-days post-cloning. Asterisk (***) corresponds to statistically significant (*p* < 0.001) difference.

Finally, as a proof of concept, we transfected CHO-K1 cells with the PvuI-linearized pcDNA3.1(+)/Luc2 = tdT plasmid. The nsPEF protocol was compared to the μsPEF protocol. The results are shown in Figure 7.

Grown G418-resistant, transfected CHO-K1 cells were checked for positive clones using fluorescence microscopy to view the fluorescent tdTomato protein. Fluorescent microscopy images of nontransfected CHO-K1 cells (CTRL) and positively pcDNA3.1(+)/Luc2 = tdT plasmid-transfected cells, using the nsPEF protocol (nsPEF), can be seen in Figure 7A. Bioluminescence detection assays were performed at day 5 and 10, using the best luminescent CHO-K1 clones received during µs and ns-pulsed electric fields’ electrotransfection protocols. The results are shown in Figure 7B.

It can be seen in Figure 7B that the CHO-K1 cell line was stably transfected with the linearized pcDNA3.1(+)/Luc2 = tdT plasmid with both protocols used. However, the bioluminescence of electrotransfected cells using nsPEF was 7-10-fold higher compared to that of the µsPEF procedure.

## 4. Discussion

The main aim of this study was to compare nano- and microsecond-range electrotransfection protocols for different cell lines (CHO-K1, 4T1) and primary murine DCs, which has not been performed before. 

It is well known that some of the main parameters affecting electroporation effectiveness are electric field strength, number of pulses, duration of pulse, frequency, molecular size, and concentration of the transported molecules [6,7,8,9,10,11,12]. In our study, two different conditions of electroporation were used: µsPEF (1.2 kV/cm × 100 µs × 8 pulses) and nsPEF (7 kV/cm × 300 ns × 100 pulses (1 MHz)). In addition, two different molecular-size plasmids (4.7 kbp p-EGFP-N1 and 8.5 kb pcDNA3.1(+)/Luc2 = tdT) were used and their optimal concentration for electrotransfection was evaluated.

To reach efficient transfection, high permeabilization of the target cells must be ensured. Both microsecond- and nanosecond-range protocols triggered high permeabilization of the CHO-K1, 4T1, and murine DCs, which indicated similar susceptibility of these cells to electroporation. The CHO-K1 permeabilization results agree with established knowledge [14,15]. So far, information about the triple-negative breast cancer 4T1 cell line and primary murine DC permeabilization using similar parameters was not reported. 

In the microsecond and nanosecond range, three concentrations of different plasmids were tested, and it was determined that the use of 0.07 μg/μL of the pEGFP-N1 and pcDNA3.1(+)/Luc2 = tdT plasmids resulted in a significantly lower transfection efficiency, compared with higher plasmid concentrations (0.2 μg/μL or 0.3 μg/μL). The results are in good agreement with available knowledge [12].

Surprisingly, the size of the plasmid did not have a significant effect on the electrotransfection of the CHO-K1 cells. It was not the case for 4T1 and DCs where the transfection rate was reduced several-fold. The differences in response could be explained by the differences in cell size and possible cell elongation during the treatment, which has a significant influence on gene transfection [41]. The acquired transfection rates using the GFP-encoding plasmid are in agreement with the available literature. Similar results of GFP-transfected 4T1 cells were reported by Flanagan et al. [42], where about a 15% transfection rate was achieved. In our study, we achieved more than 30%, which may be due to different buffers and protocols employed in the electrotransfection experiments. Our results indicate that the application of CHO-K1 cells as a model cell line is not always suitable for prediction of the transfection rates for other cells lines. However, it is perfect for comparison of various protocols between other studies.

Gene transfer into primary immune cells is of essential relevance for scientific and therapeutical applications, especially in DC vaccine studies to transfer tumor-associated antigens. One of the methods to generate genetically modified dendritic cell cancer vaccines is electroporation. The murine DC transfection efficiency was similar to the 4T1 cell line when a significant reduction in efficiency was observed with a larger plasmid. Although primary DCs are often nonacceptive of cargo delivered with external nanoparticles or biochemical formulations, we showed that nsPEFs-treated murine DCs demonstrated about a 20% transfection efficiency using the GFP reporter. The results are in agreement with Firdessa and colleagues’ study [29], where DCs and other APCs were electrotransfected using eGFP-encoding mRNA. In other DC electroporation studies, preliminary experiments were performed using human lymphoblasts isolated from the bone marrow K562 cell line. Cells were electroporated and the transfection efficiencies of eGFP-encoding mRNA and plasmid DNA electroporation were estimated [43].

Based on the literature [31], different maturations of DCs can have an effect on the electrotransfection efficiency. Nevertheless, in our study, the electrotransfection efficiency of different treated murine DCs with the GFP-encoding plasmid did not improve compared with immature DCs, which is in agreement with data by Michiels et al. [28]. In their study, immature and matured DCs were electrotransfected using eGFP-encoding mRNA and there were no significant differences in transgene expression 24 h after DC electroporation. Nevertheless, Chung et al. reported that the transfection efficiency after millisecond electroporation with enhanced green fluorescent protein (eGFP) mRNA was higher for immature than for matured DCs [44], while [31] revealed a different response pattern. The differences in response between the works could be explained by the differences in the applied pulse parameters, type of primary cells, and buffers involved. 

In our study, the effects of nsPEF were of utmost interest. Currently, long μs–ms-range pulses dominate the clinical electrotransfection protocols [45]. However, due to the short duration but high number of pulses involved, the nsPEF protocols offer better energy control to prevent Joule heating [46], ensure less oxidation due to reduced electrolysis [47,48], and enable a more homogenic treatment of heterogeneous structures [49]. In addition, due to the high-frequency component, the associated muscle contractions and potential pain can be minimized [50]. Microsecond electroporation is considered a safe and efficient methodology for drug and gene delivery; however, all those negative factors can be further optimized with a logical evolution toward the shorter-duration pulse range. The tendency is already observed in the electrochemotherapy context—the short-duration bipolar and unipolar pulses have been focused on in recent years [51,52]. This work presents a methodology of pulse compression into MHz bursts, which enables effective cells transfection, and is a significant improvement of available methods.

## 5. Conclusions

To conclude, for the first time, we have compared the transfection of CHO-K1, 4T1 cells lines, and primary murine DCs and have shown the limited applicability of the model cell line (CHO-K1) for the prediction of electrotransfection rates. It was shown that both plasmid size and cell type affect the transfection efficiency dramatically even though the susceptibility to electroporation itself is similar. For the first time, the applicability of nsPEF for the electrotransfection of DCs was highlighted and the protocols for successful electrotransfection were reported. The results of this study show the high applicability of nsPEF for the further development of optimal conditions for gene vaccination.

## Figures and Tables

**Table 1:** Viability of cells after electrotransfection normalized to untreated control. Asterisk (*) corresponds to statistically significant difference (*p* < 0.05) versus μsPEF procedure.

Cells	CHO-K1		4T1		DCs	
Treatment	μsPEF	nsPEF	μsPEF	nsPEF	μsPEF	nsPEF
p-EGFP-N1	95 ± 3%	94 ± 4%	74 ± 3%	96 ± 2% *	65 ± 15%	72 ± 8%
pcDNA3.1(+)/Luc2 = tdT	94 ± 5%	98 ± 4%	70 ± 7%	84 ± 5% *	63 ± 5%	73 ± 10%

## Data Availability

Data available from the corresponding author E.R. on request.
